# Surgical management in hepatoblastoma: points to take

**DOI:** 10.1007/s00383-022-05356-z

**Published:** 2023-01-12

**Authors:** Maciej Murawski, Viola B. Weeda, Piotr Czauderna

**Affiliations:** 1 Department of Pediatric Surgery and Urology, Medical University of Gdansk, Gdansk, Poland; 2 Department of Surgery, University Academic Medical Centre Groningen, University of Amsterdam, Amsterdam, The Netherlands; 3 Department of Surgery and Urology for Children and Adolescents, Medical University of Gdansk, 1-6 Nowe Ogrody St., 80-803 Gdansk, Poland

**Keywords:** Children, Hepatoblastoma, Liver tumour, Surgical treatment

## Abstract

Hepatoblastoma is the most common primary malignant paediatric liver tumour and surgery remains the cornerstone of its management. The aim of this article is to present the principles of surgical treatment of hepatoblastoma. All aspects of surgery in hepatoblastoma are discussed, from biopsy, through conventional and laparoscopic liver resections, to extreme resection with adjacent structures, staged hepatectomy and transplantation.

## Introduction

Hepatoblastoma (HB) is definitely a “surgical tumour”. Surgery remains the cornerstone of management and complete resection is crucial for cure [1–3]. In recent years, tremendous progress has been made in surgical armentarium and technique. This allows for complex liver resections with minimal operative morbidity and mortality. Numerous articles about surgical treatment of hepatoblastoma have been published [4–7]. In order not to duplicate previous publications, we present a concise summary of key points in HB surgery complemented by tables. The main features of HB are shown in Table 1.

**Table 1:** Key features of hepatoblastoma in children

Hepatoblastoma is the most frequent of the malignant paediatric liver tumors. HB comprises 1% of all pediatric malignancies. Its incidence is increasing by as much as 2.7% per year
HB developes usually in the absence of underlying liver disease
Typical clinical presentation: asymptomatic abdominal mass, with no associated systemic symptoms
PRETEXT (PRETreatment EXtent of Disease) system is used to stratify tumours and plan the extent of resection
Surgery remains the cornerstone of management and complete resection is crucial for cure

## Historical perspective

An overview of historical perspective relevant to the management of HB can be found in Table 2.

**Table 2:** Milestones in history of hepatoblastoma and liver tumour treatment

The first successful resection of a solid liver tumour was performed by Carl von Langenbuch in 1887 in Berlin [8]
In 1898 the first case of HB was described in the English literature, but the term “hepatoblastoma” was introduced by Willis in 1962 [1]. He defined it as “an embryonic tumour that contains hepatic epithelial parenchyma.”
In 1908 James Hogarth Pringle developed a technique to minimise blood loss during hepatic surgery by clamping the hepatic pedicle (now commonly known as the Pringle maneuver) [9]
In 1951 O'Sullivan reported a successful left hepatic lobectomy in a 5-year-old girl with hepatoma. One month after the operation the patient developed metastases and she died one year after surgery [10]
In 1954 Claude Couinaud published the report defining segmental liver anatomy, contributing majorlyed to a reduction in surgical morbidity [11]
Introduction of cisplatin- and doxorubicin-containing chemotherapy regimens in the 1980s [12]

## Biopsy—yes or no?

A diagnostic tumour biopsy is strongly recommended for all patients with a primary liver tumour. Excluded from this paradigm are benign tumours (e.g., infantile hemangioma) in the youngest children, hepatocellular neoplasms not otherwise specified (HCN-NOS; tumours previously designated as transitional liver cell tumours) in older children, and hepatocellular carcinoma (HCC) in adolescents [4]. Currently, core needle biopsy (Tru-Cut) under ultrasonographic or laparoscopic guidance is recommended. Biopsy of hepatoblastoma is safe and complications are rare (predominantly self-limiting bleeding) [4, 6, 13]. Sufficient tissue is essential for a definitive diagnosis, as pathology subtypes of HB help to determine prognosis. For instance, small cell undifferentiated (SCU) histology in HB patients is generally considered to be associated with an unfavourable outcome [14]. Even a single focus of SCU tumour in a histologically heterogenous lesion warrants stratification of the patient as high risk. However, in February 2022, a study by Trobaugh-Lotario et al. has shown contradictory results [15]. The authors analysed 35 patients enrolled on Children’s Oncology Group (COG) study AHEP0731. These patients had some elements of SCU identified on central pathological review. No adverse effect on outcome was observed in SCU group, but the presented results require confirmation. On the end of the spectrum, many studies have reported a correlation between well-differentiated fetal (WDF) histology and better outcome [16]. Unfortunately, diagnosis of WDF histology is not possible with biopsy only, or with a post-chemotherapy specimen, as it requires evaluation of the completely resected tumour before chemotherapy [17]. In 2011 in Los Angeles during an International Pathology Symposium the pathological classification of paediatric liver tumours was discussed, and a new international paediatric liver tumour consensus classification was developed [17]. Recommendations for sampling of paediatric liver tumour were also presented:

- Biopsy should be performed before chemotherapy.
- Intraoperative, rapid pathological analysis should be avoided.
- Fine-needle aspiration biopsy should be avoided for diagnosis, as it does not provide enough tissue to evaluate tumour.
- As many as five, and preferably ten, cores of tumour should be obtained, where possible, from different regions of the tumour.
- A biopsy of the adjoining normal liver should be taken for molecular tests.
- To prevent tumour seeding along the needle tract, the biopsy technique should be coaxial and the needle should be passed through “healthy” liver, which will be resected at the definite tumor resection.

## Planning of liver surgery. PRETEXT system

**Fig. 1:**
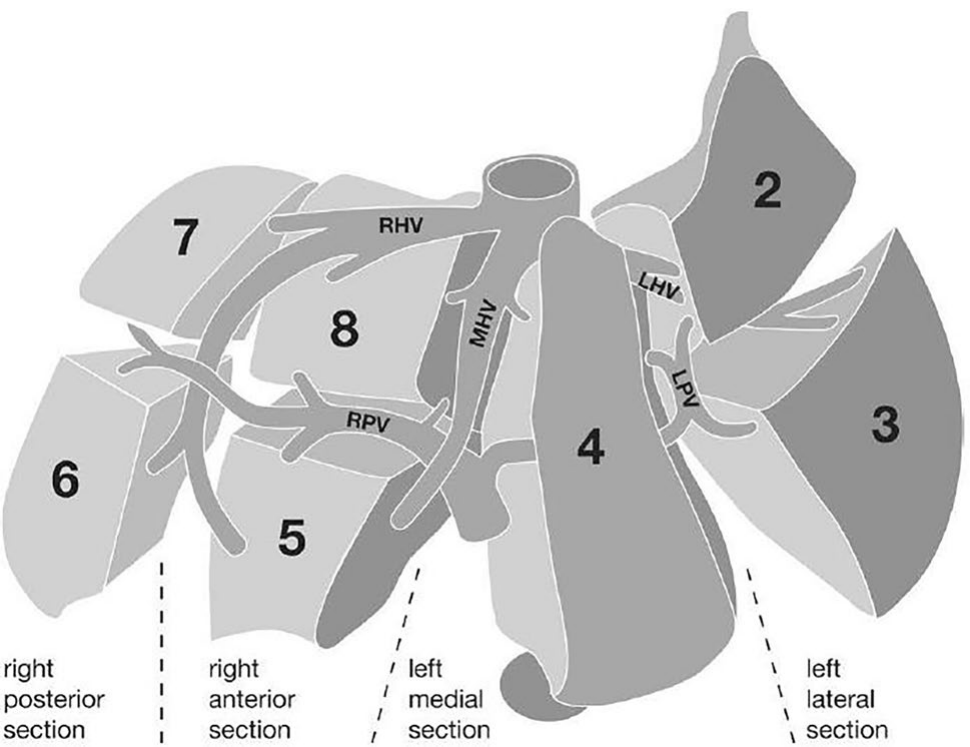
Segmental anatomy of the liver. Reprinted with permission from Derek J. Roebuck et al. Pediatric Radiology, Springer Nature [51]

**Fig. 2:**
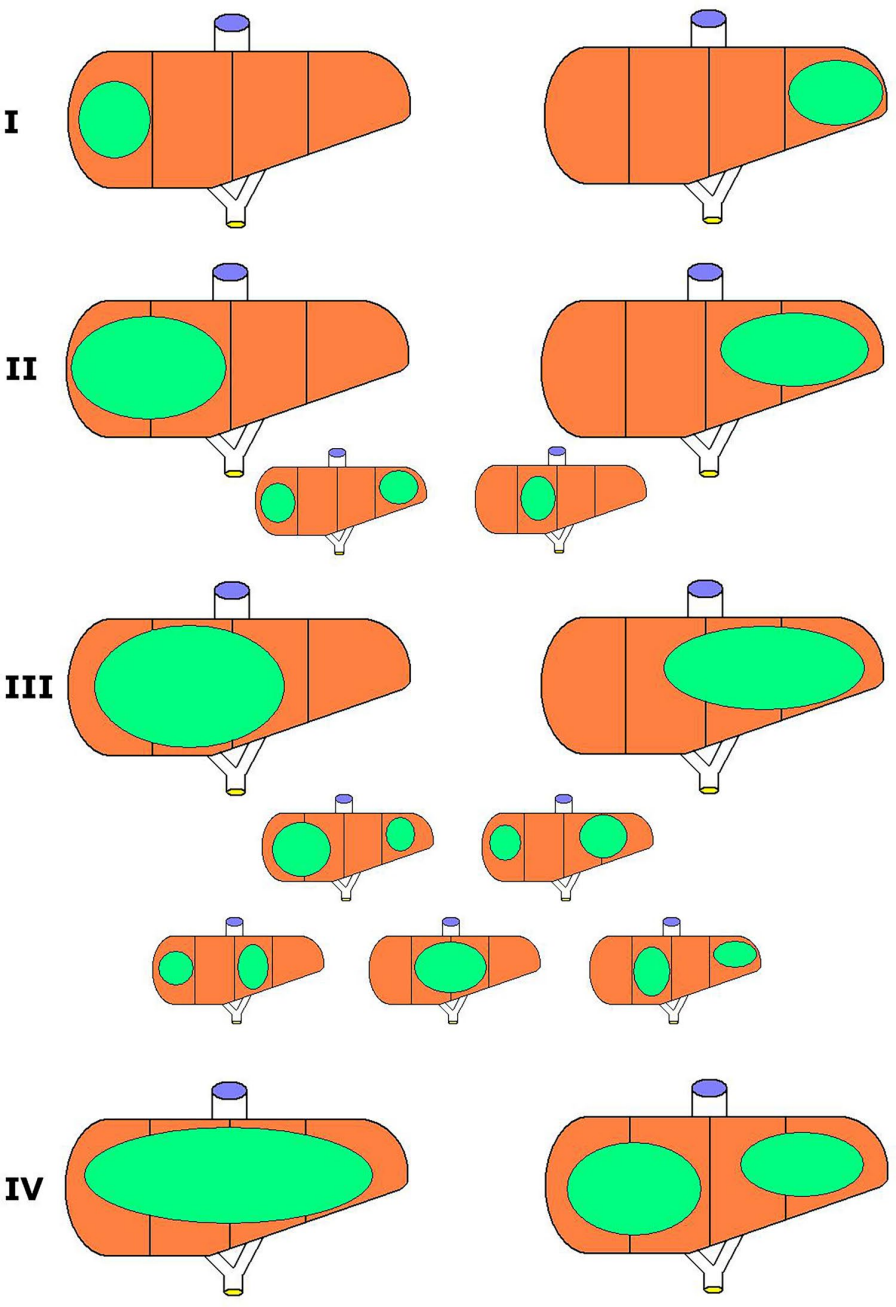
PRETEXT system (author: Maciej Murawski)

Good knowledge of liver anatomy and high quality imaging (doppler US, CT and/or MRI) are essential to assess resectability. Since its development in 1992, the PRETEXT (PRETreatment EXtent of Disease) system is used for planning of liver surgery, to predict tumour resectability and to predict prognosis. PRETEXT is based on segmental anatomy of the liver (Fig. 1). The latest PRETEXT system description was presented by Towbin et al. in Pediatric Radiology in February 2018 [18]. The PRETEXT system is depicted in Fig. 2 and definitions are described in detail in Table 3 and presented in Fig. 2. Important remarks regarding PRETEXT system:

- Cavernous transformation of the main portal vein is classified as (evidence of) tumour thrombus.
- Extrahepatic disease is a rare situation, occuring in less than 5% of patients with HB. Simple ascites is not considered extrahepatic disease.
- Multifocal tumours are present in 20% of patients with HB.
- Rupture of the tumour during surgery is not considered tumour rupture.
- Lymph node metastases are uncommon in HB and require pathologic confirmation.
- HB metastastses occur most commonly to the lung; this happens in 20% of HB cases. Biopsy is not necessary for diagnosis, because it is unusual for other lesions to mimic metastases.
- Tumours close to hilar structures leading to compression of local structures may lead to classification challenges. Tumours that are pushing vascular structures aside may cause pressure changes that can mimic invasion on imaging [19].

**Table 3:** PRETEXT system (based on: 2017 PRETEXT revision by Alexander J. Towbin et al.)

	Definition
PRETEXT group	
I	1 section involved
	3 contiguous sections are tumour free
II	1 or 2 sections involved
	2 contiguous sections are tumour free
III	2 or 3 sections involved
	1 contiguous section is tumour free
IV	4 sections involved
Annotation factors	
V	Venous involvement. V-positive tumour: (1) tumour obliterating^a^ or encasing^b^ (> 50% or 180^O^) all 3 hepatic veins or IVC, (2) tumour thrombus in any one hepatic vein or IVC^c^
P	Portal venous involvement. P-positive tumour: (1) tumour obliterating or encasing (> 50% or 180^O^) both portal veins or main portal vein, (2) tumour thrombus in either or both the right and left portal veins, or the main portal vein
E	Extrahepatic disease contiguous with the main liver tumour
F	Multifocality. Two or more hepatic tumours surrounded by normal liver tissue
R	Tumour rupture. Free fluid in the abdomen or pelvis at diagnosis with 1 or more findings of haemorrhage on imaging: (1) septations within fluid, (2) high-density fluid on CT (> 25 HU), (3) blood on MRI, (4) visible rupture/hepatic capsular defect. Clinical findings of haemorrhage: HCT < 25%, HGB < 7 g/dl, blood pressure drop, requiring blood transfusion, acute abdominal signs
C	Caudate: Involvement of the caudate lobe (segment 1) – the tumour is at least PRETEXT II
N	Lymph node metastases: (1) Lymph node with a short-axis diameter of > 1 cm or a portocaval lymph node > 1.5 cm, (2) spherical lymph node with loss of fatty hilum. Definitive involvement of lymph nodes should be confirmed histologically
M	Distant metastases. M-positive: One pulmonary nodule ≥ 5 mm, or 2 or more nodules, each ≥ 3 mm in diameter

^a^Obliterating—tumour is compressing the vein so that the lumen is not visible, ^b^Encasing—tumour is touching and surrounding the vein by more than 50% or 180°

^c^IVC—inferior vena cava

## Tumour resection

### The timing and extent of surgical resection. Primary or delayed surgery?

Traditionally, the traditional American (COG) approach has been laparotomy at diagnosis with an upfront resection in all patients. According to the International Paediatric Liver Tumour Study Group ‘SIOPEL’, the convention was to treat all patients with neoadjuvant chemotherapy and perform delayed resection [6]. In order to reach consensus and establish a common "international" approach, leaders from the four cooperative trial groups (SIOPEL, Children’s Oncology Group, the German Society for Paediatric Oncology and Haematology, and the Japanese Study Group for Paediatric Liver Tumours) joined forces to form the CHIC consortium (the Children’s Hepatic tumours International Collaboration). CHIC created a single database containing the information about 1605 children treated in eight multicentre hepatoblastoma trials over 25 years. Novel prognostic factors for hepatoblastoma were identified and established factors were confirmed. Identified risk factors include: PRETEXT group, age at diagnosis, AFP level and the presence of a PRETEXT annotation factor [20, 21]. This was used to create a common international risk stratification system and served as a groundwork for global, prospective study (the Paediatric Hepatic International Tumour Trial, PHITT). In this trial, patients are staged into four risks groups: Very low risk (Group A), low risk (Group B), intermediate risk (Group C), and high risk (Group D). In addition to the HB risk groups, there are also two groups for HCC. The PHITT protocol may be found online: https://www.birmingham.ac.uk/Documents/collegemds/trials/crctu/phitt/Protocol/Current/PHITT-Protocol-version-3-0-17Oct2018.pdf*.*

Currently **resection at diagnosis** is recommended for tumours that are categorized as very low risk. This applies to the following cases: PRETEXT I and II, M-, resectable at diagnosis (VPEFR-), and additionally in PRETEXT II: age < 8 years, AFP > 100. For other tumours, timing of resection is less straight forward. Surgical resection is performed after satisfactory evaluation based on imaging after neoadjuvant chemotherapy. An upfront resection is recommended only when a segmentectomy or nonextended hemihepatectomy with at least 1 cm margin is possible on middle hepatic vein and/or main portal vein division, and there is no concern for macrovascular involvement [4, 5].

### General principles and basic techniques of liver resection (Table 4)

**Table 4:** General remarks about HB surgery

The size of the tumour alone is not a contraindication to resection
Tumour resectability depends upon surgical expertise
Knowledge of liver anatomy, experience in liver surgery and specialised equipement are absolutely necessary
The goal of surgery is to achieve complete tumour resection with negative margins. Incomplete macroscopic tumour resection is associated with worse outcome!
Anatomic resections are usually recommended

#### Types of liver resections

**Fig. 3:**
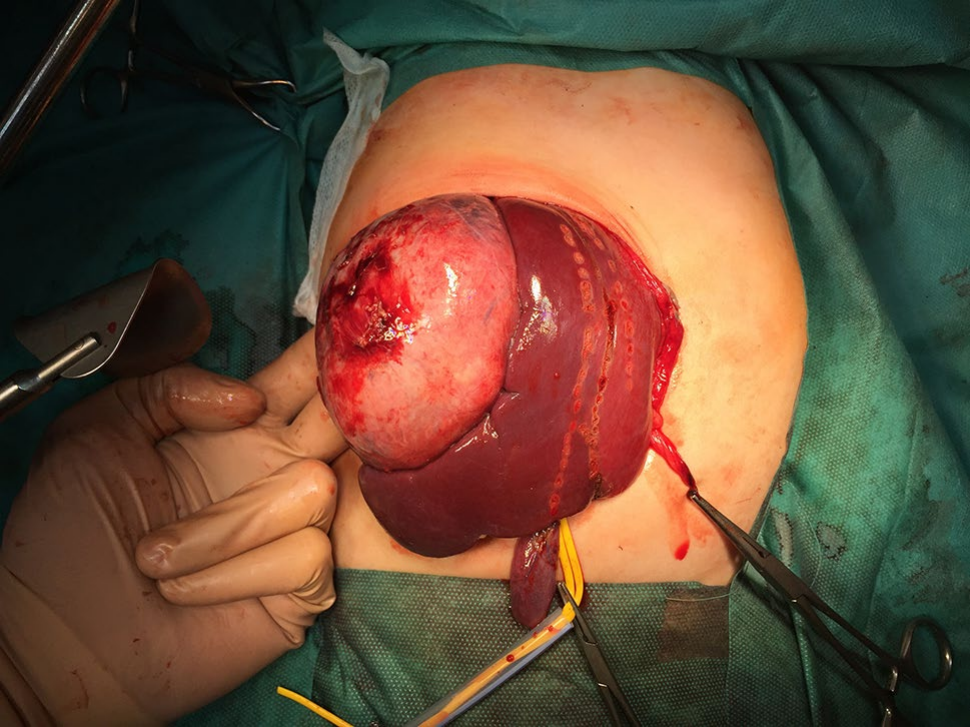
Tumor of the right hepatic lobe. Intraoperative view showing ischemic delineation of the right liver

Both knowledge of liver anatomy and experience in liver surgery are absolutely necessary to decide on the type of liver resection. Anatomic resections (based on Couinaud’s division of liver anatomy) are generally recommended (segmentectomy, hemihepatectomy) (Fig. 3). The type of resection depends on response to preoperative chemotherapy, pre-existing liver disease, size of the tumour and the remnant liver volume. A liver remnant that is too small for the patient’s size will increase the risk of postoperative liver failure (please refer to VII.ALPPS) [22]. Nomenclature of liver resections is presented in Table 5. Atypical, non-anatomic, wedge resections are associated with worse outcome [23] and are justified only infrequently, usually in multifocal tumours, when LTX is contraindicated due to metastatic disease. However, the basis for these recommendation are German HB89 and HB94 studies performed 20 years ago. Qureshi et al. reported 25 nonanatomic liver resections and compared the results with 95 anatomic resections [24]. He concluded that nonanatomic liver resection is feasible with no positive margins in carefully selected patients and performed by surgeon well experienced in liver surgery. The rate of complications and outcomes was similar. More studies are needed to revise the guideline for liver resection in hepatoblastoma.

**Table 5:** Nomenclature of liver anatomy and resections based on: The Brisbane 2000 Terminology of Liver Anatomy and Resections

Anatomical term	Couinaud segments	Term for surgical resection
Right (hemi)liver	5–8 (± seg.1)	Right hepatectomy/right hemihepatectomy (± seg.1)
Left (hemi)liver	2–4 (± seg.1)	Left hepatectomy/left hemihepatectomy (± seg.1)
Right anterior section	5.8	Right anterior sectionectomy/sectorectomy
Right posterior section	6 and 7	Right posterior sectionectomy/sectorectomy
Left medial section	4	Left medial sectionectomy/segmentectomy 4
Left lateral section	2 and 3	Left lateral sectionectomy/bisegmentectomy 2,3
Right hemiliver + left medial section	4 and 5–8 (± seg.1)	Right trisectionectomy/extended right hemihepatectomy/ extended right hepatectomy
Left hemiliver + right anterior section	2–5 and 8 (± seg.1)	Left trisectionectomy/extended left hemihepatectomy/ extended left hepatectomy
Segments 1–8	Any one of seg.1–8	Segmentectomy
2 contiguous segments	Any two of seg.1–8	Bisegmentectomy

#### Stages of liver resection

**Fig. 4:**
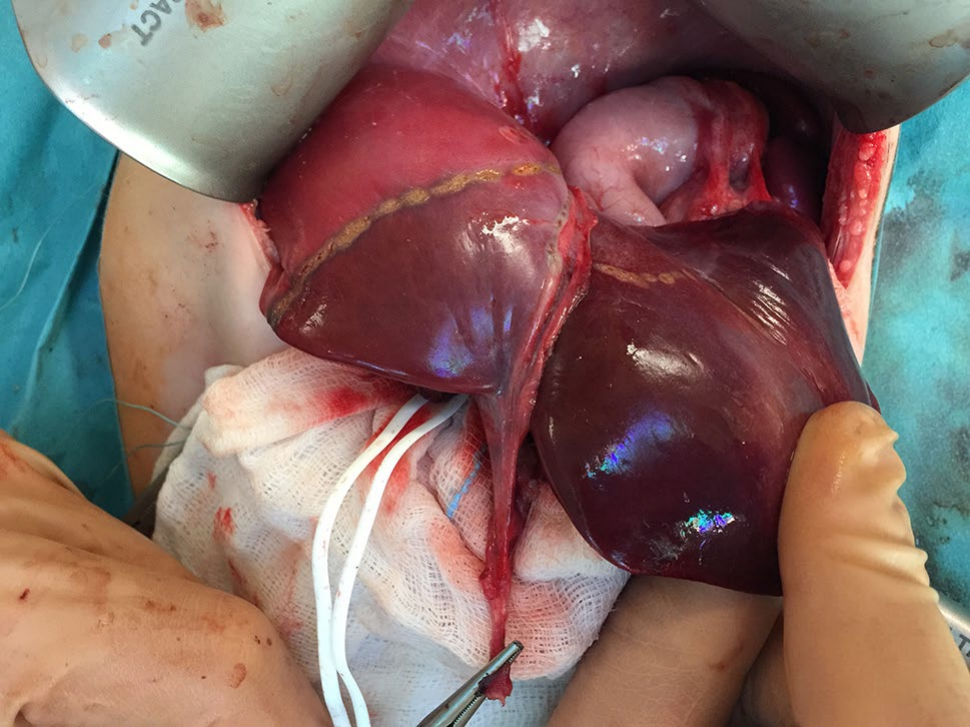
Intraoperative view showing ischemic delineation of the left liver

**Fig. 5:**
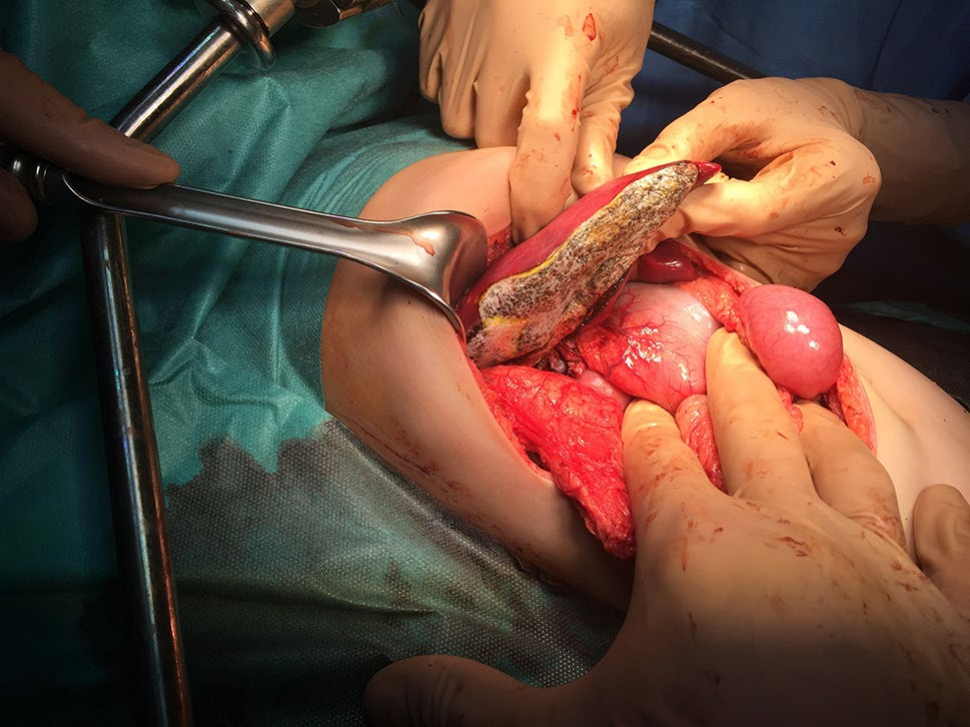
TachoSil placed on the liver surface after hepatic resection

- Liver mobilisation. Triangular ligament ligated and transected, and the falciform ligament is incised until the subdiaphragmatic inferior vena cava (IVC) is reached. To mobilise the right lobe, the right triangular ligament is incised. On the left side, the left triangular ligament is transected.
- Intraoperative ultrasonography. It is very important to evaluate the resection margin from the point of view of oncological safety, particularly in the case of extensive tumour or multifocal lesions. It allows to reveal liver anatomy, locate lesions, and define tumour connections with portal pedicles and hepatic veins.
- Inflow control (hilar phase) (Fig. 4). The arterial and portal venous blood supply to the part of the liver to be removed can be controlled by extrahepatic or intrahepatic pedicle ligation. Knowledge of the anatomy of the portal vessels is crucial. The portal triad is composed of common hepatic duct, portal vein and hepatic artery. The arterial and portal venous blood supply to the part of the liver to be removed can be controlled by extrahepatic or intrahepatic pedicle ligation. Knowledge of the anatomy of the portal vessels is crucial. In children, the standard technique is to divide the hepatic artery and portal vein separately although mass transection with a stapler can be used, too. Control of the relevant biliary pedicle may accompany vascular dissection, but there is a risk of biliary injury. To avoid this, the biliary structures can be secured during parenchymal transection.
- Outflow control (venous phase). Extrahepatic isolation of the hepatic veins is possible in most cases. This technique allows for good control in case of haemorrhage during the next phase. In some situations hepatic veins can be transected during parenchymal transection. This particularly applies to the middle hepatic vein (MHV), as it is often involved in the surgical margin.
- Parenchymal transection. After inflow and outflow control, a clear line of ischemia is visible and parenchymal dissection is proceeded along this line. Methods of parenchymal transection are, inter alia: (1) finger or clampfracturing the tissue, (2) harmonic scalpel, (3) ultrasonic energy (Cavitron Ultrasonic Surgical Aspirator, CUSA), (4) radiofrequency energy (the salinelinked radiofrequency dissecting sealer), (5) water-jet dissection, (6) the application of surgical stapler. To minimize blood loss the Pringle maneuver (portal triad clamping) can be applied. It is worth remembering that intermittent clamping is better tolerated by the liver remnant than continuous occlusion [25]. When performed in intervals the portal triad is usually clamped for 10–15 min and unclamped for 3–5 min. This allows for a longer potential total occlusion time [22].
- Oozing control. After cutting the liver surface it can be secured with bipolar coagulation, LigaSure, argon beam, clips, and/or various topical hemostatic agents (Fig. 5).

### Complete tumour resection—is it still the gold standard?

It is well-known (and mentioned above), that the goal of surgical resection is to achieve complete tumour clearance, however, the healthy liver tissue margin required in paediatric HB is a matter of debate [5]. The traditionally recommended 1 cm margin of normal liver parenchyma is sometimes difficult to achieve especially in young children, and probably is not required. The question may arise whether a smaller margin (for example, a few milimeters) will be sufficient for cure. Also, surgical margins are sometimes judged as microscopically tumour positive by pathologists. To evaluate the influence of a microscopically positive resection on prognosis, Aronson et al. analysed patients from SIOPEL-2 and -3 studies and compared 58 children with microscopically positive margin and 371 completely resected children [26]. No differences in the local recurrence rate, event free survival, and overall survival were observed between SR and HR groups at 5 years of follow up at least in the setting of cisplatin neoadjuvant therapy. How can these results be explained? Firstly, the positive margin on the tumor side does not necessarily mean the presence of tumour cells on the patient side. Instruments used for parenchymal dissection—such as the CUSA, which in its course may literally suck part of the margin away, and haemostatic treatments (see above) may be responsible for tumour cell clearance on liver remnant surface. Secondly, the postoperative continuation of very effective platinumbased chemotherapy may eliminate micro residuals. This is the first formal analysis related to the effect of microscopically positive margin on the outcome of hepatoblastoma patients. The presented results require confirmation. For this reason, complete resection continues to be the gold standard and should be always encouraged. Answer to the above mentioned questions may be given by the analysis of the large series from the PHITT study.

## Laparoscopic liver resection

Over the last decade, laparoscopic liver resections for paediatric hepatic tumours have been successfully performed, but most laparoscopic hepatectomies reported are case presentations and small case series of nonanatomical resections for small, peripheral and usually benign, isolated lesions [27]. There is only one larger study on the subject in the current English literature published by Veenstra and Koffron in 2015 [28]. They performed 36 resections—15 were for benign tumours and 21 were for malignant tumours (20 hepatoblastomas and 1 fibrolamellar HCC). Of the 31 purely laparoscopically performed resections, there were 10 segmentectomies, 5 sectionectomies, and 16 hemihepatectomies. The contemporary acceptable indication for laparoscopic liver resection in adult patients is the presence of a single lesion measuring 5 cm in diameter or less located in liver segments 2 to 6 (so called “laparoscopic segments”) [29]. In general, laparoscopic liver resection in children is feasible and safe but

- Patients must be carefully selected.
- Specific training is needed and the accompanying learning curve should be taken into account (experience in both open hepatobiliary and laparoscopic surgery are crucial).
- The rules of safe oncological surgery must always be followed.

## ALPPS (associating liver partition with portal vein ligation for staged hepatectomy)

This new treatment option for patients with marginally resectable liver tumours was introduced in 2007, but formally described only in 2011 [30]. As large resections are connected with the risk of postoperative liver dysfunction, the success of the resection is based on a suitable future liver remnant (FLR). Unfortunately, there is no clear definition of FLR in children and different limits were applied across the studies: FLR/TLV (total liver volume) < 15%, < 25%, < 30%, < 40% and FLR < 1.5% of body weight. [31] ALPPS allows for rapid hypertrophy of the FLR, and therefore, helps to avoid postoperative hepatic insufficiency [32].

### Stages of ALPPS

- First operation: (1) portal vein ligation, (2) in situ splitting of the liver parenchyma (partial partition—dissection to the level of the MHV, total partition—dissection to the IVC), (3) eventual clearance of the FLR from satellite neoplastic lesions in case of multifocal tumour.
- Second operation: (1) transection of the hepatic artery and the bile duct, (2) transection of the hepatic vein, (3) appropriate part of the liver is removed.

### Advantages

- Rapid hypertrophy of the future liver remnant—47–93% within 7–14 days [32].
- Increased likelihood of possible R0 resection in selected cases.
- Avoidance of postoperative liver failure.
- Reduced the interval between the 2 surgeries, and thus less chance of tumour progression.

### Disadvantages

- ALPPS may promote tumour growth, but the mechanism is unclear [32] and there is lack of sufficient data to prove this thesis.
- Rapid hypertrophy does not always correlate with sufficient liver function [32].

Fortunately, children tolerate major resections better than adults (in children the FLR should be at least 25%), and therefore ALPPS is rarely necessary in this age group. The first (and the last so far) series of paediatric patients treated with ALPPS was presented by Wiederkehr et al. in 2015. There were two patients with HB, 1 with HCC, 1 with RMS, and 1 with FNH [33].

## Extreme liver resections and with resection of adjacent organs/structures

Cases of HB involving three or four sectors of the liver (POSTTEXT III–IV) or the hilum of the liver may be cured by an extended (right or left) liver resection or a total hepatectomy and LTX including partial hilar vessel resection and reconstruction [34, 35]. When the tumour—often as tumour thrombus—extends into the vena cava and/ or the right atrium and does not clear with chemotherapy, resection through combined laparotomy and sternotomy with use of preoperative cardiopulmonary bypass/ extracorporeal membrane oxygenation may be successful [36–38]. Furthermore, in rare instances complete resection of the tumour may involve resection of part of the diaphragm, stomach, omentum, pancreas or spleen in addition to a partial liver resection or in addition to a complete hepatectomy and LTX [37]. In a larger series of 27 patients with POST-TEXT III or IV who underwent extended liver resections with or without resection of adjacent structures, 5-year OS was 81% while 5-year EFS was 62% [37]. Several cases of successful multivisceral transplant (MVT) for hepatoblastoma extending into the portomesenteric vessels have been described as well [39, 40]. In the series of Lee et al. two patients with hepatoblastoma undergoing MVT are described; after 4.5 and 8 years of follow up respectively there was no evidence of tumour recurrence.

- Cases of HB warranting extreme resections and vascular reconstructions, should be carried out in transplant centres of excellence in paediatric liver surgery.
- We recommend a combination of a surgeon well experienced in liver surgery and a liver transplant surgeon in the operating team for such HB cases.

## Liver transplant for hepatoblastoma

- 10–20% of all HB cases require liver transplant (LTX) [41]
- Indications and contraindications for liver transplant for hepatoblastoma are presented in Table 6
- Locally advanced tumours are a challenge for surgeons.

**Table 6:** Indications and contra-indications for liver transplant for hepatoblastoma

	Comments
Indications	
Multifocal PRETEXT IV	No active extrahepatic tumour sites (metastases or regional extension)
Solitary PRETEXT IV	Potential downstaging to PRETEXT III after neoadjuvant chemotherapy
	If resection possible: only in very experienced hands
PRETEXT III with major vascular involvement (P + , V +)	Unresectable tumour after neoadjuvant chemotherapy
Central tumours involving segments IV, V, VIII in close proximity to major vessels (main PV, PV bifurcation, hepatic veins)	Possible central hepatectomy (mesohepatectomy, middle lobectomy)^†^
Tumours adjoining major vessels	If aggressive resection possible: only in very experienced hands
Tumours invading major vessels	Resection is very risky (bleeding, tumour residual, compromise of vascular inflow/outflow)
Contra-indications	
Lung metastases or regional extension not completely cleared during preoperative chemotherapy and not resectable	Microscopic foci of chemoresistant tumor highly probable

*P + * portal venous involvement, *V + * venous involvement, *PV* portal vein

^†^It is very rare to have an indication for these procedures

In certain cases, it may represent quite a challenge to decide whether to perform an extreme or complex resection or have the patient undergo a transplant [5]. Continuing chemotherapy when the tumour remains unresectable is contraindicated. It is better to avoid toxic effects of intense chemotherapy. Additionally, there is a risk of induction of chemotherapy resistance [42, 43]. Whether to operate and in which way must ideally be decided after no more than four cycles of chemotherapy. Interestingly, Lovvorn has shown that the biggest hepatoblastoma response to induction therapy occurs during first two cycles. He proposed to shift the timing of this decision (resection/LTX) after cycle No. 2 of induction therapy [43]. Hence, it is definitely better to refer complex HB cases to a transplant center early in the course of treatment.

- Some reports have questioned the role of salvage LTX (performed for local relapse or in case of incomplete tumour resection), suggesting that it is connected with inferior survival when compared with primary LTX (80% vs. 30–40%), although there are some conflicting studies [44–47].
- It is important to note that hepatoblastoma patients who present with extrahepatic or metastatic active disease at diagnosis that fully clears with chemotherapy and/or surgery are still candidates for transplantation.
- Survival rates after primary transplantation are excellent: about 80–85% 5 years OS [46].
- However, it should be borne in mind that LTX has its own “dark side”: a relatively high complication rate leading to comorbidity and the need for immunosuppressive drugs and their side effects such as secondary neoplasms.

## Preoperative tumour rupture

Spontaneous rupture of HB is very rare and occurs in 3–9% of HB [48]. The diagnosis is based on clinical signs (blood pressure drop, acute abdominal signs), laboratory findings (HCT < 25%, HGB < 7 g/dl) and haemorhage signs as well as liver capsule violation on imaging (see also Table 3). Control of the bleeding can be achieved by transcutaneous arterial embolization (TAE) or surgically with primary/delayed resection. It is very important to avoid massive blood loss (both due to tumour rupture and during the resection). On the one hand, massive blood loss and shock may result in ischemic injury to the liver resulting in post-hepatectomy liver failure [48]. On the other hand, evidence from adultstudies suggests that blood transfusions have a negative impact on survival and time to recurrence [49]. Furthermore, tumour rupture carries the risk of intra-abdominal tumour seeding, however, there is no solid data to support this notion [48].

## Resection of pulmonary metastases—before or after primary tumour resection?

The most common location of distant metastases in HB is the lung (occurring in 20% of cases). The timing of metastasectomy is currently under discussion [50–52]. The traditional approach is as follows:

- Children with resectable HB and synchronous lung metastases: pulmonary metastasectomy should be performed after the resection of the primary tumor, because the control of primary HB is associated with improved outcomes. After hepatectomy, pulmonary metastasectomy is usually preceeded by 1 or 2 chemotherapy courses.
- Patients with an indication for LTX: pulmonary metastases that persist after chemotherapy should be resected before transplantation.

An open question is whether chemotherapy alone is enough to clear the lungs in patients undergoing LTX. The potential need for surgical exploration to confirm the clearance of metastases is discussed in the literature [50].

## The main hazards of hepatic resection (Table 7)

**Table 7:** Surgical complications of liver resection

Surgical complication	Most common cause	Comments
Bleeding	Intra- and postoperative haemorrhage	Potentially life-threatening How to avoid this: (1) Meticulous oozing control at the end of resection if necessary with coagulant agents (2) Avoid aggresive dissection near large vessels
Intraoperative cardiac arrest (incidence 1–2%)	Massive blood loss Air embolism	Good communication beetween surgeon and the anesthesiologist, for instance about timing of required low central venous pressure (parenchymal dissection), occurrence of bleeding, and signs of disturbed coagulation Application of PEEP (Positive End-Expiratory Pressure) during vein and IVC dissection
Bile leakage (incidence 4–17%)	Bile duct injury at the level of the hilum Bile leakage from the cut surface	Definition: an increased bilirubin concentration (at least 3 times greater than serum bilirubin concentration) in the intra-abdominal fluid (drain) Avoid non-anatomic resections Sometimes drainage with Roux-en-Y limb of jejunum is necessary
Post-hepatectomy liver failure	Small liver remnant Vascular flow disturbance Bile duct obstruction Viral infection Severe septic conditions	Depending on the etiology Liver transplantation may be needed
Infection	Surgical site infection and wound dehiscence Pneumonia Hepatic or perihepatic abcess Cholangitis Peritonitis	Optimise anabolic state preoperatively Use antibiotic prophylaxis and repeat if the surgery takes > 6 h Use meticulous fascial suturing technique Optimise postoperative pain management and keep intubated period to a minimum Use respiratory physical therapy for post operative respiratory rehabilitation Be meticulous in postoperative follow up and consider draining larger abcesses with a low threshold Treat signs of cholangitis aggresively and promptly Perform urgent imaging and if indicated do not delay reoperation
Other	Adhesive bowel obstruction Pleural effusion	No definitive measures to avoid these complications are known

- Liver resection is a high-risk but “safe” operation with a mortality rate less than 5% in experienced hands [45, 46]
- Morbidity rates remain high and range from 4 to 56% [53–55]

## Unresectable and recurrent hepatoblastoma

When the tumour remains unresectable after chemotherapy various therapeutic approaches may be applied. These include liver transplantation, extreme resection, staged hepatectomy (see ALPPS section), and/or interventional radiology procedures (transarterial radioembolization, transarterial chemoembolization) [56, 57]. Relapses after HB treatment are quite rare and the treatment for recurrent HB is not standardised. Semeraro et al. analysed the group of relapsed HB patients treated in the SIOPEL 1–3 studies [58]. The data are presented in Table 8. The therapeutic options for relapsed HB are chemotherapy with or without surgical resection, liver transplantation and thermal ablation (radiofrequency ablation—RFA, microwave ablation, cryoablation) [13, 59]. Unfortunately, there are no clear criteria for selection of the appropriate method of locoregional therapy. Only a few publications describe the use of RFA in children [60, 61], but it seems that RFA is a valid therapeutic option, which may even lead to cure in highly selected relapsed cases.

**Table 8:** Relapses in hepatoblastoma SIOPEL 1–3 patients

Recurrences	59/695 (8.4%)	
Time to relapse from diagnosis	12 months (4–115 months) Late relapse (> 3 years)—6 patients	
Site of relapse	Local	21 (36%)
	Metastatic	32 (55%)
	Combined	5 (9%)
	Unknown	1
Site of metastases	Lungs	27
	Peritoneum	4
	Central nervous system	1
Treatment	Chemotherapy	21
	Chemotherapy + surgery	25
	Surgery	7
	Palliative care	5
Chemotherapy regimens	Carboplatin + etoposide	13
	Carboplatin + etoposide + doxorubicin	6
	Irinotecan	12
	High-dose cyclophosphamide	6
Resection	Local relapse	16 (including 1 LTX)
	Lung metastasectomy	15
	Peritoneal implants	1
Survival	23 patients (39%) (18 in CR2 and 5 in CR3) are alive with no evidence of disease	
3-year OS/EFS	43%/34%	

## Data Availability

The data that support the findings of this study are available from the corresponding author upon reasonable request.
